# Determinants of preterm birth among reproductive age women in sub-Saharan Africa: Evidence from the most recent Demographic and Health Survey data-2019-2022

**DOI:** 10.1371/journal.pone.0305810

**Published:** 2024-06-25

**Authors:** Berhan Tekeba, Masersha Asmare Techane, Belayneh Shetie Workneh, Alebachew Ferede Zegeye, Almaz Tefera Gonete, Medina Abdela Ahmed, Yilkal Abebaw Wassie, Mulugeta Wassie, Alemneh Tadesse Kassie, Mohammed Seid Ali, Enyew Getaneh Mekonen, Tadesse Tarik Tamir, Sintayehu Simie Tsega

**Affiliations:** 1 Department of Pediatrics and Child Health Nursing, School of Nursing, College of Medicine and Health Sciences, University of Gondar, Gondar, Ethiopia; 2 Department of Emergency and Critical Care Nursing, School of Nursing, College of Medicine and Health Sciences, University of Gondar, Gondar, Ethiopia; 3 Department of Medical Nursing, School of Nursing, College of Medicine and Health Sciences, University of Gondar, Gondar, Ethiopia; 4 School of Nursing, College of Medicine and Health Sciences, University of Gondar, Gondar, Ethiopia; 5 Department of Clinical Midwifery, School of Midwifery, College of Medicine and Health Sciences, University of Gondar, Gondar, Ethiopia; 6 Department of Surgical Nursing, School of Nursing, College of Medicine and Health Sciences, University of Gondar, Gondar, Ethiopia; University of Cambridge, UNITED KINGDOM

## Abstract

**Introduction:**

Preterm birth is the leading cause of both infant and neonatal mortality. It also had long-term consequences for the physical and neurological development of a growing child. The majority of these and related problems occur in low- and middle-income countries, particularly in sub-Saharan Africa, due to resource scarcity to sustain the lives of premature babies. Despite this, there is a paucity of recent information on the pooled prevalence and factors associated with preterm birth in sub-Saharan Africa. Therefore, this study aimed to update the pooled prevalence and determinants of preterm birth in sub-Saharan Africa based on the most recent Demographic and Health Survey data.

**Methods:**

A cross-sectional study design using the most recent demographic and health survey data from eight sub-Saharan African countries was used. We included a total weighted sample of 74,871 reproductive-aged women who gave birth in the five years preceding the survey. We used a multilevel logistic regression model to identify associated factors of preterm birth in sub-Saharan Africa. The adjusted odds ratio at 95% Cl was computed to assess the strength and significance of the association between explanatory and outcome variables. Factors with a p-value of <0.05 are declared statistically significant.

**Results:**

In this study, the pooled prevalence of preterm birth among reproductive-aged women in eight sub-Saharan African countries was 3.11% (95% CI: 2.98–3.25). Working mothers (AOR = 0.61; 95% CI: 0.38–0.97), being married (AOR = 0.63; 95% CI: 0.40–0.99), and having media exposure (AOR = 0.59; 95% CI: 0.36–0.96) decrease the odds of preterm birth. On the other hand, being low birth weight (AOR = 17.7; 95% CI: 10.7–29.3), having multiple pregnancies (AOR = 3.43; 95% CI: 1.82–6.45), having a history of terminated pregnancies (AOR = 1.56; 95% CI: 1.01–2.41), being un-educated (AOR = 3.16; 95% CI: 1.12–8.93), being of a maternal age above 35 (AOR = 1.63; 95% CI: 1.08–2.45), maternal alcohol use (AOR = 19.18; 95% CI: 13.6–38.8), and being in the low socio-economic status (AOR = 1.85; 95% CI: 1.11–3.07) of the community increase the odds of preterm birth.

**Conclusion:**

The burden of preterm birth among reproductive-age women in sub-Saharan Africa showed improvements as compared to previous findings. To further lessen the burden, policymakers and other pertinent organizations must prioritize maternal health, expand media access, educate and empower women, and promote a healthy lifestyle for reproductive-age women.

## Introduction

Preterm birth refers to the birth of a baby between 20 to 37 weeks of gestation [1]. Globally, an estimated 13.4 million babies were born preterm in 2020 [2]. Preterm birth complications are the leading cause of under-five and infant mortality [3, 4]. More than half of preterm births and the majority of their complications and deaths occur in Asia and sub-Saharan Africa [5]. Across countries, the rate of preterm birth ranges from 4–16% in 2020. Three-quarters of these deaths could be prevented with correct, cost-effective interventions [6].

Preterm births mostly happen spontaneously, but some are brought on by diseases or problems throughout pregnancy related to infections, multiple pregnancies, and chronic conditions like diabetes and high blood pressure [2, 4]. Preterm birth is the leading cause of neonatal mortality and is associated with long-term physical, neuro-developmental, and socio-economic effects [7]. Preterm babies are substantially more likely to have adverse birth outcomes like respiratory distress syndrome [7, 8], broncho-plumonary dysplasia [9], necrotizing entro-colitis [10, 11], neurodevelopmental complications [12], cerebral palsy [13], chronic lung disease [14], glucose intolerance [15], and hypertension [16, 17] than those born at term.

According to prior study findings revealed across the globe, several factors are associated with preterm birth. Accordingly, maternal age [18], maternal education [19], maternal substance use [20], marital status [21], residence [22], wealth index [23], mode of delivery [24], birth interval [25, 26], history of miscarriage and stillbirth [27], multiple pregnancy [28, 29], and ANC visit [30, 31] significantly associated with preterm birth.

The World Health Organization (WHO) recommends tried and true methods to lower preterm pregnancy and its complications, including having an antenatal care visit (ANC) visit, counseling healthy diets, ultrasound screening of gestation, and multiple pregnancies [32]. More than three-quarters of preterm births could be saved with feasible, cost-effective care, and further reductions are possible through intensive neonatal care [33]. Despite this, there is a huge disparity in the survival of premature babies depending on where they are born. Even for these born in a health clinic or hospital, essential newborns are often lacking in SSA. More than 90% of extreme preterm births born in low-income countries die within the first few days of life, yet less than 10% of extremely preterm babies die in high-income settings [6]. An up-to-date assessment of the burden of the problem and the priority predictors shall be done to inform concerned bodies on how to combat the burden of prematurity and its complications in these resource-limited settings.

It is well established that preterm birth has both immediate and long-term effects for the growing baby related to prematurity and limited neonatal care units in low-income nations like the SSA. Thus, frequent assessments of the burden of prematurity and its associated morbidity and mortality at the multi-country level help relevant authorities make decisions accordingly on premature birth and its adverse consequences across countries. However, there is a paucity of recent information on the pooled prevalence and factors associated with preterm birth in sub-Saharan Africa. Therefore, this study aimed to update the pooled prevalence and determinants of preterm birth in sub-Saharan Africa based on the most recent Demographic and Health Survey data, which helps inform relevant authorities to tackle the problem.

## Method

### Data source and sampling procedure

We used the most recent Demographic and Health Survey data to determine the pooled prevalence and determinants of preterm birth among reproductive-age women in Sub-Saharan Africa (SSA), which were conducted in eight sub-Saharan African (SSA) countries between 2019 to 2022. Every five years, the DHS surveys are routinely conducted in low- and middle-income countries using standardized, pretested, and validated questionnaires. In order to enable multi-country analysis, the survey adheres to a comparable standard approach for the creation of questions, sampling, data collection, and coding.

A stratified, two-stage cluster sampling technique is used in the DHS surveys. The first step was the random selection of clusters or EAs that encompass the entire country from the sampling frame derived from the most recent national survey that was made accessible. In the second step, interviews were held in a subset of the target population’s households using systematic sampling, which was applied to all of the households mentioned in each cluster (women aged 15–49). A weighted sample of 74,871 reproductive-age women who gave birth within the five years prior to the survey in each country was included in this study. For women who had given birth more than once in the five years before the survey, the most recent birth was taken into account. Additionally, women in their reproductive years who had a missing value for the outcome variable were not included in the study.

### Study design, period

A population-based cross-sectional study was conducted using a secondary analysis of data from 2019 to 2022 in eight sub-Saharan African countries. We used the most recent DHS data from 2019 and onwards to update the prevalence of preterm birth in the study area. The Demographic and Health Survey was a cross-sectional survey that examined population health with a focus on maternal and child health, as well as population health indicators of global interest.

### Study and source population

The source population consisted of all babies born prematurely in the five years of the survey period across eight sub-Saharan African countries. The study population consisted of babies born prematurely in the five years preceding the survey period in the selected enumeration areas (EA) in the respective countries. Enumeration is the primary sampling unit of the survey clusters.

### Inclusion criteria and exclusion criteria

Preterm births born in countries that had a DHS report since 2019 in sub-Saharan Africa were included. Sub-Saharan African countries that had not reported DHS in the specified period of time (since 2019) were excluded from the study.

### Study variables

The study outcome variable was preterm birth. Preterm birth was determined by using the DHS variable "b20," which denotes the duration of pregnancy. The duration of pregnancy was available as 5–10 months in the DHS data. These women-related variables were collected during the DHS survey by using computer-assisted personal interviews. Then we re-categorize the duration of pregnancy as below and above 9 months. Those babies born before 9 months (5 to 8 months) were considered “preterm” and coded as "1." Those babies born at 9 months and above were considered a “term” and coded as "0.”

### Independent variables

Both individual and community-level factors were reviewed from different literatures, and these include maternal age, child sex, pregnancy intention, birth interval, Caesarean section delivery, maternal educational level, media exposure, place of delivery, twin pregnancy, ANC visit, mother-work status, birth weight, maternal BMI, contraceptive use, maternal height, maternal history of alcohol and cigarettes use, history of terminated pregnancy, and household wealth quintiles. Residence, distance to a health facility, community ANC utilization, community women’s education, community media exposure, and community wealth status were community-level factors aggregated from individual-level factors.

Wealth index were categorized as poor, middle, rich [34]. Plurality refers to multiple pregnancies. ANC use was classified as optimal if a mother had more than 4 visits during her pregnancy and non-optimal if the mother had less than 4 ANC visits during her pregnancy [35].

Maternal education status was categorized as no formal education, primary, secondary, and higher in our study and other EDHS [36].

Media exposure was aggregated from three variables in the DHS data set. These variables are: 1) frequency of reading a newspaper or magazine; 2) frequency of listening to radio; and 3) frequency of watching television. These three variables each have three responses. a. not at all; b. less than once a week; c. at least once a week. Then, we merged the above three variables as one variable, and we categorized not at all responses as “having no media exposure” and less than once and at least once a week as “having media exposure" [37].

Distance to a health facility was available in DHS data as a questioners: “getting medical help for self (distance to a health facility),” and responses were “a big problem” or “not a big problem." Those who responded to the above question as having a big problem, we consider them to have “distance difficulty,” and those who responded as not having a big problem, we consider them to have “distance difficulty.”

Birth weight was categorized as small for a weight less than 2500 g, normal for a weight of 2500–3900 g, and large for a weight greater than 4000 g.

Maternal BMI was categorized as low for BMI<18.49 kg/m^2^, normal for 18.5–24.9 kg/m2, and high for ≥25 kg/m^2^.

### Data management and model selection

After being extracted from the EDHS portal, Stata version 14 was used to enter, code, clean, record, and analyze the data. In DHS data variables are nested by clusters and those within the same cluster show more similarities than those with separate clusters. Thus, using the traditional logistic regression model violates the assumptions of independent observation and equal variance across clusters. Therefore, a more sophisticated model is required to account for cluster characteristics. A multi-level multivariable logistic regression model was employed in the study to identify the variables associated with preterm birth. In the analysis, four models were fitted. The first (null) model, which contains only the outcome variables, assesses the degree of intra-cluster variation in preterm birth. The second model contains individual-level variables; the third model contains only community-level variables; and the fourth model contains both individual-level and community-level variables [38]. A p-value of 0.05 was used to define statistical significance. Adjusted odds ratios with corresponding 95% confidence intervals (CIs) were calculated to identify independent predictors of preterm birth.

Variation of the outcome variable or random effects was assessed using the proportional change in variance (PCV), intra-class correlation coefficient (ICC), and median odds ratio (MOR) [39, 40]. To quantify the variation between clusters, the proportionate change in variance (PCV), median odds ratio (MOR), and intra-class correlation coefficient (ICC) were calculated. Using clusters as a random variable, the ICC shows how preterm birth varies amongst clusters and is calculated as: ICC = V_C_/ (V_null_ + 3.29) ×100%, where V_c_ is the variance of the cluster and V_null_ is the variance of the null model. The MOR is the median value of the odds ratio between the areas with the highest and lowest risk of preterm birth, and the calculated as: MOR = _e_^0.95√Vc^; where, V_c_ is the variance of cluster. Furthermore, the PCV determines how different factors account for variations in the prevalence of preterm birth and is computed as PCV = (V_null_-V_C_)/V_null_ ×100%, where V_C_ is the cluster-level variance and V_null_ is the variance of the null model [41, 42]. The likelihood of preterm birth and independent variables at the individual and community levels were estimated using the fixed effects analysis. An adjusted odds ratio (AOR) and 95% confidence intervals with a p-value of 0.05 were used to evaluate it and display its strength. Due to the hierarchical nature of the model, models were compared using deviation = -2 (log likelihood ratio), and the best-fit model was determined by taking the model with the lowest deviance. By calculating the variance inflation factors (VIF), the variables employed in the models were checked for multi-collinearity; the results were within acceptable ranges of one to ten [43–45].

## Ethics approval and consent to participate

This study is a secondary analysis of the DHS data, so it does not require ethical approval. For conducting our study, we registered and requested the datasets from DHS, which were publically available, and received approval to access and download the data files. According to the DHS report, all participant data were anonymized during the collection of the survey data. More details regarding DHS data and ethical standards are available online at http://www.dhsprogram.com.

## Result

In this study, eight (8) sub-Saharan African countries were included. Since most African countries did not have recent demographic and health survey data, we only included eight countries with the most recent demographic and health survey data (2019–2022) (**Table 1)**.

**Table 1:** Country, sample size and survey year of Demographic and Health Survey included in this study.

County	Sample size	Survey year
Gabon	3,255	2020/21
Gambia	12,805	2019
Kenya	29,906	2022
Liberia	2,911	2019/20
Rwanda	4,130	2019/20
Serra Leon	10,106	2019
Senegal	6,254	2019
Tanzania	5,504	2022
Total	74,871	2019–2022

## Socio-demographic, maternal and child characteristics

This study comprised a weighted sample of 74,871 reproductive-age women who had delivered within the five years preceding the survey in each respective country. Nearly two-thirds (65.11%) of mothers reside in rural areas. More than two-thirds of mothers were aged 20–34; one-fourth were aged above 35 years, whereas about 5.8% were aged below 20. More than two-thirds (66.75%) of mothers were currently married. One-third (32.84%) of mothers didn’t have formal education. Nearly half (49.1%) of preterm were females, and half (50.9%) were males. Regarding the obstetric history of participants, the majority (94.3%) of women had an ANC visit (94.3%). A significant proportion (15.16%) of participants had a history of terminated pregnancies. The majority (91.75%) of women deliver by normal vaginal delivery. The majority of participants (84.94%) deliver in health facilities. More than two-thirds (71.07%) of participants had exposure to the media. More than one-third (38.33%) of participants were contraceptive users **(Table 2)**.

**Table 2:** Socio-demographic, maternal, obstetric and child related factors.

Variables	Response	Frequency	Percentage
**Socio-demographic characteristics**			
Maternal education	Un-educated Primary Secondary Higher	24,589 25,034 21,016 423	32.84 33.44 28.07 5.65
Maternal age	<20 years 20–34 years ≥35 years	3,880 51,528 19,463	5.8 68.82 26.00
Marital status	Married Un-married	49,973 24,898	66.75 33.25
Husband education	Un-educated Primary Secondary Higher	23,483 17,912 15,281 5,501	37.77 28.81 24.58 8.85
House hold head sex	Male Female	56,062 18,809	74.88 25.52
Mother currently working	No Yes	32,298 42,573	43.14 56.86
Media exposure	No Yes	21,662 53,204	28.93 71.07
**Child related characteristics**			
Child sex	Male Female	38,113 36,758	50.90 49.10
Birth weight	Low Normal High	3,732 33,626 3,736	6.52 58.70 6.53
Birth interval	≤2 years >2 years	11,302 45,488	19.90 80.10
Place of delivery	Home Health facility	10,530 64,341	14.06 85.94
Deliver by CS	Yes No	4,663 51,807	8.25 91.75
**Maternal, obstetric and clinical related factors**			
Plurality	Single Multiple	72,217 2,654	96.46 3.54
Terminated pregnancy	No Yes	63,518 11,353	84.84 15.16
Maternal BMI	<18.5 18.5–24.9 ≥25	2,921 21,453 11,635	8.11 59.56 32.30
Pregnancy c/c	No Yes	2,703 5,676	32.74 67.26
Pregnancy wanted	Yes No	59,010 3,836	93.90 6.10
Post natal check	No Yes	55,211 19,660	73.74 26.26
ANC check	Non-optimal Optimal	2,902 45,747	5.97 94.03
Maternal height	<150 cm 150–155 cm ≥155	2,711 6,902 26,413	7.35 18.72 71.66
Anemia	Yes No	8,774 12,645	40.96 59.04
Contraceptive use	Use Not use	28,699 46,172	38.33 61.77
Mothers smoke	Yes No	9,458 50,820	16.3 83.77
Mother use alcohol	Yes No	870 20,180	4.13 95.87
**Community level factors**			
Place of residence	Urban Rural	26,126 48,745	34.89 65.11
Region	Central East West	6,376 30,090 38,405	8.52 40.19 51.29
Community illiteracy	Low High	42,488 32,423	56.69 43.31
Community ANC use	Low High	27,075 47,789	36.17 63.83
Distance to health facility	Big problem Not a big problem	22,958 40,501	36.18 63. 82
Community media exposure	Low High	34,862 40,009	46.56 53.44
Community poverty level	Low High	39,803 35068	53.16 46.84

ANC: Antenatal care; BMI: Body mass index; C/C; complication CS: Cesarean section

### The pooled prevalence of preterm birth among reproductive age women in SSA

The pooled prevalence of preterm birth among reproductive-age women in eight SSAs was 3.111% (95% Cl; 2.98–3.25). Our finding is lower than the previous study in sub-Saharan Africa, which was 5.33%, and the WHO recent report across the globe, which ranged from 4–16% [32, 46].

### Random effect and model analysis

In the null model, the ICC indicates that 33.43% of the total variability for preterm birth was due to differences between clusters in enumeration areas, with the remaining 66.57% attributable to individual differences. In addition, the median odds ratio also revealed that preterm was heterogeneous among clusters. According to the null model, if women were chosen at random from two clusters in the enumeration areas, women in the higher cluster would have a 3.39 times higher chance of giving preterm birth as compared to women within clusters of lower risk. Regarding PCV, about 33% of the variability in preterm was explained by the final full model (model III). Model III was selected as the best-fitting model because it had the lowest deviance (**Table 3**).

**Table 3:** Random effect and model fit statistics of preterm birth among reproductive age.

Parameter	Null Model	Model I	Model II	Model III
Variance	1.65	1.14	1.29	1.10
ICC	33.43	25.75	28.23	25.06
MOR	3.39	2.76	2.95	2.71
PCV	reference	30.95	0.22	0.33
LLR	-9025.4	-588.52	-6460.57	-583.35
Deviance	18050.8	1,177.03	12921.14	1,166.67

ICC: Intra cluster correlation coefficient MOR: median odds ratio PCV: proportional change in variance LLR: log likelihood ratio

### Factors associated with preterm birth

In the final model (model III) of multivariable multilevel logistic regression, both individual and community-level factors were significantly associated with preterm birth. Accordingly, maternal education, maternal age, twin pregnancy, history of terminated pregnancy, low birth weight, maternal history of alcohol use, marital status, media exposure, working status of the mother, and community poverty level were significant variables associated with preterm birth.

Women with no formal education were 3.6 times (AOR = 3.16; 95% CI: 1.12–8.93) more likely to deliver a preterm baby as compared to women with higher education. Mothers aged 35 years and older had a 63% (AOR = 3.16; 95% CI: 1.08–2.45) higher chance of delivering preterm birth as compared to mothers aged 20–34 years. Women’s having a history of terminated pregnancy were 56% (AOR = 1.56; 95% CI: 1.01–2.41) more likely to deliver preterm birth as compared to women who had no history of terminated pregnancy. Kids born with a low birth weight were 17 times (AOR = 17.7; 95% CI: 10.7–29.3) more likely to be delivered prematurely as compared to kids with a normal birth weight. Women who used alcohol during pregnancy were 19 times (AOR = 19.18; 95% CI: 13.6–38.8) higher in delivering preterm birth as compared to women with no alcohol use during pregnancy. Women from high community poverty levels were 85% (AOR = 1.85; 95% CI: 1.11–3.07) more likely to deliver preterm babies as compared to women from low community poverty levels. On the other hand, married women were 37% (AOR = 0.63; 95% CI: 0.40–0.99) less likely to deliver a preterm baby as compared to unmarried women. Women from media-exposed households were 41% (AOR = 0.59; 95% CI: 0.36–0.96) less likely to deliver a preterm baby as compared to households with no media exposure. Currently, working mothers are 39% (AOR = 0.61; 95% CI: 0.38–0.97) less likely to deliver preterm birth as compared to non-working mothers. **(Table 4)**.

**Table 4:** Multivariable multi-level logistic regression analysis of individual and community level factors associated with preterm birth in sub-Saharan Africa; 2019–2022.

Individual and community level factors	Responses	Model I AOR (95% Cl)	Model II AOR (95% Cl)	Model III AOR (95% Cl)
**Demographic related characteristics**				
Maternal education	No education Primary Secondary Higher	3.16 (1.11–8.94) 1.18 (0.46–3.00) 1.16 (0.47–2.83 1		3.16 (1.12–8.93)* 1.52 (0.93–2.48) 1.13(0.63–2.03) 1
Maternal age	<20 year 20–34 >35	2.04 (0.4–10.34) 1 1.62(1.08–2.45)		2.14(0.43–10.77) 1 1.63 (1.08–2.45)*
Marital status	Married Unmarried	0.61 (0.39–0.96) 1		0.63 (0.40–0.99)* 1
Husband education	No education Primary Secondary Higher	1.12 (0.44–2.87) 1.18 (0.46–3.00) 1.16 (0.47–2.83) 1		1.08 (0.42–2.74) 1.20 (0.47–3.06) 1.10 (0.45–2.68) 1
House hold head sex	Male Female	0.64 (0.41–1.01) 1		0.66 (0.42–1.04) 1
Mothers current working	Working Not working	1 0.62 (0.39–0.97)		1 0.61 (0.38–0.97)*
Media exposure	Yes No	0.56(0.34–0.91) 1		0.59 (0.36–0.96)* 1
**Child related factors**				
Newborn sex	Male Female	0.64 (0.41–1.01) 1		0.87 (0.60–1.27) 1
Birth interval	≤ 2 years >2 years	1.10 (0.65–1.86) 1		1.13 (0.67–1.92) 1
Birth weight	Low Normal High	18.1(10.96–29.9) 1 0.50 (0.15–1.66		17.7 (10.7–29.3)* 1 0.52 (0.16–1.73)
Place of delivery	Home Health-facility	0.54 (0.28–1.04) 1		0.54 (0.28–1.04) 1
Deliver by cesarean section	Yes No	1.10 (0.59–2.06) 1		1.09(0.58–2.03) 1
**Maternal obstetrics and clinical related factors**				
Plurality	Yes No	3.48 (1.84–6.5) 1		3.43 (1.82–6.45)* 1
Terminated Pregnancy	Yes No	1.56 (1.01–2.41) 1		1.56 (1.01–2.41)* 1
Maternal BMI	<18.5 kg/m2 18.5–24.9 kg/m2 ≥25 kg/m2	0.91(0.37–2.27) 1 1.15(0.77–1.73)		1.69 (0.87–3.29) 1.17 (0.72–1.88) 1
Wanted pregnancy	Yes No	0.60 (0.28–1.30) 1		0.60 (0.27–1.30) 1
PNC check	Yes No	1 0.77 (0.51–1.15**)**		1 0.77 (0.52–1.15)
ANC check	Non optimal Optimal	4.43 (1.02–19.34) 1		
Maternal height	<150 cm 150–155 cm ≥155	1.68(0.87–3.27) 1 1.17(0.72–1.89)		1.69 (0.87–3.29) 1 1.17(0.72–1.88)
Mother anemic	Yes No	1.05 (0.71–1.55) 1		0.98 (0.69–1.39) 1
Contraceptive use	Use Not use	1.39 (0.94–2.05) 1		1.39 (0.93–2.06) 1
Mothers smoke	Yes No	1.28 (0.28–5.85) 1		1.39 (0.31–6.27) 1
Mothers alcohol use	Yes No	13.06 (3.39–57.8) 1		19.18(13.6–38.1)* 1
**Community level factors**				
Place of residence	Urban Rural		1 0.63 (0.55–0.72)	1 0.7 (0.46–1.08)
Community illiteracy	High Low		0.52 (0.42–0.64) 1	0.84(0.56–1.26) 1
Community ANC use	Low High		1 1.15(0.93–1.42)	1 1.09 (0.73–1.63)
Distance to health facility	Big problem Not a big problem		0.95 (0.84–1.08) 1	0.97 (0.66–1.40) 1
Community media exposure	Low High		1 1.91 (1.53–2.38)	1 1.35 (0.9–2.01)
Community poverty level	High Low		1.35 (1.10–1.66) 1	1.85 (1.11–3.07)* 1

*: Significantly associated (p<0.05)

## Discussion

Prematurity is the primary cause of death for children under five worldwide. Half of premature babies in low-income settings die from a lack of cost-effective, affordable care, such as warmth, breastfeeding support, and basic treatment for infection and asphyxia. Identifying and reducing avoidable determinants of preterm birth is a first step towards improving neonatal survival. The priority in low-income countries like sub-Saharan Africa should be preventing avoidable causes of preterm birth and its adverse consequences; thus, up-to-date information is needed to inform relevant authorities [32].

This study revealed that the pooled prevalence of preterm birth among reproductive-age women in SSA was 3.11% (95% Cl; 2.98–3.25). Our finding is lower than the previous study in sub-Saharan Africa, which was 5.33%, and the WHO recent report across the globe, which ranged from 4–16% [6, 46]. This could be explained by the fact that the healthcare system, ANC utilization, and focus on mothers and newborns have been enhanced and strengthened in many countries across Africa [47]. Furthermore, nations may endeavor to accomplish the sustainable development goal of reducing maternal and child mortality. Therefore, countries should adopt the WHO strategy and recommendations on avoidable preventive mechanisms for preterm birth, such as ANC visits, early newborn care, and outfitting and facilitating available neonatal care intensive units, in order to lower and reduce the burden of preterm birth and its complications.

This study found factors significantly associated with preterm birth. Accordingly, maternal current work status, marital status, media exposure, birth weight, multiple pregnancies, history of terminated pregnancy, maternal educational status, maternal age, maternal alcohol use, and socio-economic status of the community are significantly associated with preterm birth.

According to this study, we found higher odds of preterm birth among non-educated mothers as compared to mothers with a higher education. This is supported by studies done across the globe [19, 48–50]. This could be explained by the fact that women with less education tend to engage in unhealthy behaviors and have worsening health conditions. However, well-educated mothers might lead healthy lifestyles and have good psychosocial wellbeing. Additionally, among women who experience educational disparity, it is linked to a number of unfavorable situations. Moreover, uneducated mothers have less knowledge regarding the available health services and how to utilize them, so they will miss antenatal care visits [50–52].

The likelihood of preterm birth among women aged 35 and above was found to be higher as compared to optimal maternal age (20–34) [53]. This is supported by other studies [53, 54]. Even though the exact pathway for the causation of preterm birth and advanced maternal age is yet unknown, a possible explanation could be that studies support advanced maternal age or that mothers of higher maternal age had a higher spontaneous preterm than younger mothers [55, 56]. In addition, there are hypotheses that link advanced maternal age and preterm birth, including hormonal theory that elaborates hormonal levels, including progesterone decline, with maternal age. One study supports that women diagnosed with progesterone deficiency during the luteal phase treated with progesterone had a lower rate of preterm birth than women without treatment [57].

Mothers with a history of terminated pregnancy had a higher chance of giving preterm birth as compared to women with no history of pregnancy termination. This is supported by studies done in different parts of the world [58, 59]. The association between preterm birth and a history of aborted pregnancy may be explained by shared biological, genetic, and environmental factors related to subsequent births; this implies the subsequent births had a higher tendency to be preterm [60]. Terminations of pregnancy have different consequences for subsequent deliveries, including preterm birth and congenital malformation [61]. Hence, women who have a history of terminated pregnancies should be alert to their subsequent pregnancies. Those mothers should have strict ANC follow-up, optimal nutrition, and a healthy lifestyle.

Being a twin had a higher chance of being preterm as compared to a singleton pregnancy. This is supported by the studies done in different parts of the world [62–65]. This is due to the fact that the physiology of labor begins sooner in multiple pregnancies due to greater baby sizes and higher levels of placental and amniotic fluid than in a singleton pregnancy [28]. Additionally, it could be the result of the association between multiple pregnancies and other comorbidities, including preeclampsia, antepartum hemorrhage, premature rupture of membrane, and poly-hydraminos, which increase the risk of premature birth [66]. Therefore, pregnant women should attend a health facility as soon as possible to take the required precautions against undesired pregnancy-related complications, such as preterm delivery, and plurality must be established early through ultrasound.

The chance of delivering a preterm baby was higher among mothers who consumed alcohol as compared to their counterparts. This finding was supported by studies done in different parts of the world [67–71]. Although the precise process causing premature birth in alcohol users is yet unknown, there are a few possible possibilities. First, women who drink alcohol frequently get pregnant unknowingly and then decide to use over-the-counter drugs to terminate their pregnancy, which causes them to deliver prematurely [72, 73]. Second, there is an enhanced prostaglandin level in alcohol drinkers, and a high prostaglandin level induces labor and preterm birth [74]. Third, heavy parental alcohol exposure can result in impaired fetal development, making preterm birth inevitable [75, 76].

This study found that high community poverty levels increase the odds of preterm birth as compared to rich communities. This is supported by studies done in different parts of the world [77–81]. This may be explained by the fact that mothers from low-income backgrounds had a restricted amount of food and nutrients available to developing fetuses, which results in intrauterine growth restriction (IUGR). Fetuses with IGUR were more likely to be delivered prematurely [81]. In addition, poor socioeconomic status has an impact on the psychosocial welfare of the community, resulting in poor birth outcomes [82]. For instance, poor communities live a stressful life. High levels of stress are associated with stress hormone release and inflammatory reactions; these cascades induce labor, and the growing fetus ends up with preterm birth [83]. Moreover, illicit drug use was also higher among low-socioeconomic communities, which further leads to adverse birth outcomes, including preterm birth [84]. Furthermore, the co-existence of other significant risk factors such as medical co-morbidities, a lack of prenatal care, and adverse behaviors (smoking and alcohol use), which occur more commonly in women of low socio-economic status [85].

Consistent with other studies, low-birth-weight babies had a higher chance of being preterm as compared to babies with a normal birth weight. This is supported by a study done in the United Arab Emirates, USA [86, 87]. Preterm birth is the principal determinates of low birth weight with unknown etiology [87]. This could be explained by both low birth weight and preterm birth occurring as adverse birth outcomes simultaneously. Although prematurity is a major reason for babies being born with low birth weight, low birth weight is an imperfect surrogate for preterm birth [88].

According to this study, the chance of delivering a preterm baby was lower for mothers who had media exposure as compared to women who didn’t have media exposure. This is supported by a study done in Asia [89], in which mass media exposure is positively associated with maternal health care utilization. Mothers with media exposure will have knowledge on pregnancy complications and adverse birth outcomes. Besides, media exposure increases women’s access to knowledge and improves their ability to seek health care [90].

Women who are currently not working had a lower chance of delivering a preterm baby as compared to women who were working. This is explained by research done in different parts of the world. This could be due to spontaneous preterm delivery, which might happen to mothers with excessive physical work and standing in contrast to their counterparts [91–93]. Besides, stay-at-home moms will have more time for leisure, less psychosocial stress, meal preparation and maintaining a healthy lifestyle, all of which may contribute to the good health of the unborn child. Having a job may generate income for mothers and their households, but it can limit mothers’ access to health care [94].

According to this study, married mothers had a lower chance of delivering preterm babies than their counterparts [21, 95]. This finding is supported by a previous study [96]. This may be connected to married women’s higher healthcare utilization, low psychological stress levels, and strong financial security, all of which either directly or indirectly influence the risk of premature birth [97]. In addition, people who are married have a greater sense of responsibility to engage in health activities.

This research has potential implications for health care programmers, mothers, and relevant authorities who are working for the better health of women and children by providing current and up-to-date evidence. Thus, increasing access and exposure to media, taking work leave, and forming a stable relationship lowers the risk of preterm birth. Furthermore, educating women, early identification of multiple pregnancies, and advice on abstaining from a sedentary lifestyle also lower the incidence of preterm birth.

## Conclusion

The study found that preterm birth in sub-Saharan Africa showed a decline as compared to global reports, but the problem still persists. In order to prevent preterm birth and its complications, countries and relevant authorities should give special attention to vulnerable women’s and children, educate women, improve household wealth status, strengthen media use, and perform health promotion on healthy pregnancy and lifestyle.

### Strength and limitation

The strength of this study was a multi-country survey with weighted pooled national sample data; thus, it has the potential to help programmers and policymakers develop effective interventions at the multi-country level. But it has limitations. Due to the cross-sectional nature of the data, it cannot establish a cause-and-effect relationship, and the DHS is primarily dependent on respondents’ self-reports, so there is a chance of recall bias like duration of pregnancy in our study. In addition, in this study, we included only a few countries from sub-Saharan Africa, which leads to underrepresentation. Moreover, medical causes of preterm birth like maternal hypertension, gestational diabetes, and infections are not included in the DHS data, which further lowers trustworthiness. Furthermore, in our study, the duration of pregnancy was determined in months, but as WHO recommendations and medical literature measure preterm in weeks rather than months, there might be bias. As a result, future clinical studies shall be conducted incorporating more countries, with gestation directly detected by ultrasound in weeks and adding the clinical cause of preterm birth.

## Supporting information

S1 File (ZIP)

## Abbreviations

ANC: Antenatal care
APH: Ante partum hemorrhage
EA: Enumeration area
EDHS: Ethiopian Demographic Health Survey
ICC: Inter cluster correlation
LR: logistic regression
MOR: Median Odds Ratio
SSA: Sub-Sahara Africa
PCV: Proportional Change in Variance
WHO: World Health Organization
